# Hemodialysis-Associated Problems to Solve: Current and Future

**DOI:** 10.1155/2014/382170

**Published:** 2014-03-17

**Authors:** Fumihiko Hinoshita, Ryoichi Ando, Rumi Sakai, Satoru Kuriyama

**Affiliations:** ^1^Department of Nephrology, National Center for Global Health and Medicine, Tokyo 162-8655, Japan; ^2^Department of Nephrology, Japanese Red Cross Musashino Hospital, Tokyo 180-8610, Japan; ^3^Sakairumi Clinic, Kobe 658-0046, Japan; ^4^Division of Kidney and Hypertension, Department of Internal Medicine, Jikei University School of Medicine, Tokyo 105-8461, Japan

Hemodialysis (HD) is now an indispensable medical treatment for patients with end-stage renal disease (ESRD). The early establishment of this epoch-making treatment was very difficult. Based on the original concept of blood purification in the 19th century, many great scientists and physicians, such as John J. Abel, Georg Haas, Willem Kolff, Frederik Kiil, and Belding H. Scribner, made tremendous efforts to realize safe and efficient dialysis for patients in the 20th century. Thanks to Dr. Scribner and many other enthusiastic pioneers, the method of modern HD has been established and spread throughout the world in the 1960s. Since that time, HD-related technology and drugs have greatly evolved in many ways, and it may appear that HD is a completed medical technique which would hardly need any further innovation in the 21st century.

We must take a step back and ask ourselves if HD is truly an established treatment. Is there no need for further innovation? Are there no HD-associated clinical problems? We believe nearly all nephrologists and HD-devoted physicians and medical staff as well as clinical researchers would answer “no.” Even though HD care has greatly progressed, more innovations and further modifications for HD are still necessary to improve the prognosis of HD patients and to prevent various types of complications. Moreover, some refined methods, techniques, and solutions to improve HD might be currently carried out only in limited or localized hospitals and HD facilities in particular areas. It is easy to imagine that most of these methods are not yet widely known, and they have not been introduced or tried in other hospitals or HD facilities in other countries, particularly in emerging and developing countries where HD was just introduced a mere 5 or 10 years ago and is not spread to every area of each country.

This special issue was planned and proposed to identify the current HD-associated problems which are not well known or not yet resolved. A number of researchers in clinical practice have indicated various HD-associated problems from diverse perspectives. Such problems include urogenital, cardiac, muscular, and gerontological problems as well as anemia, multiple myeloma, blood access, and also information related to erythropoietin and anticoagulant therapy. Treatment by exercises, a new hemodiafiltration (HDF) method, and so on are further included. All of these newly published findings would not necessarily ensure a perfect resolution for the problems in the HD area or nephrology; however, we hope that such contributions will lead to useful solutions and novel developments to overcome various insuperable HD-associated problems in the future. We also expected some innovative reports on CKD-mineral bone disease (MBD), HD session frequency and length, and the use of herbal or alternative medicines, all of which have been very important and still are a matter of controversy. Unfortunately, however, the important findings on these problems could not be published in the current special issue. We earnestly hope that these problems will be covered and actively discussed in a near future issue of this journal.

